# Collagen Complexity Spatially Defines Microregions of Total Tissue Pressure in Pancreatic Cancer

**DOI:** 10.1038/s41598-017-10671-w

**Published:** 2017-08-30

**Authors:** Michael D. Nieskoski, Kayla Marra, Jason R. Gunn, P. Jack Hoopes, Marvin M. Doyley, Tayyaba Hasan, B. Stuart Trembly, Brian W. Pogue

**Affiliations:** 1 Thayer School of Engineering at Dartmouth, Hanover, NH 03755 USA; 2 Department of Surgery, Geisel School of Medicine at Dartmouth, Hanover, NH 03755 USA; 3 Department of Electrical and Computer Engineering, University of Rochester, Rochester, NY USA; 4 Wellman Center for Photomedicine, Massachusetts General Hospital, Harvard Medical School, Boston, MA 02114 USA

## Abstract

The poor efficacy of systemic cancer therapeutics in pancreatic ductal adenocarcinoma (PDAC) is partly attributed to deposition of collagen and hyaluronan, leading to interstitial hypertension collapsing blood and lymphatic vessels, limiting drug delivery. The intrinsic micro﻿-regional﻿ interactions between hyaluronic acid (HA), collagen and the spatial origins of mechanical stresses that close off blood vessels was investigated here. Multiple localized pressure measurements were analyzed with spatially-matched histochemical images of HA, collagen and vessel perfusion. HA is known to swell, fitting a linear elastic model with total tissue pressure (TTP) increasing above interstitial fluid pressure (IFP) directly with collagen content. However, local TTP appears to originate from collagen area fraction, as well as increased its entropy and fractal dimension, and morphologically appears to be maximized when HA regions are encapsulated by collagen. TTP was inversely correlated with vascular patency and verteporfin uptake, suggesting interstitial hypertension results in vascular compression and decreased molecular delivery in PDAC. Collagenase injection led to acute decreases in total tissue pressure and increased drug perfusion. Large microscopic variations in collagen distributions within PDAC leads to microregional TPP values that vary on the hundred micron distance scale, causing micro-heterogeneous limitations in molecular perfusion, and narrows viable treatment regimes for systemically delivered therapeutics.

## Introduction

Pancreatic ductal adenocarcinoma (PDAC) is often associated with aggressive localized invasion of micrometastases and resistance to systemically delivered therapeutic agents. At least part of the resistance to systemic agents comes from an enhanced desmoplastic reaction within the PDAC itself, which occurs when pancreatic stellate cells (PSC) are activated from quiescent to myofibroblast-like phenotypes. This change allows for the proliferation and migration of PSC’s and the secretion of extracellular matrix (ECM) components^1^, such as hyaluronic acid and collagen^2^. It is this dense ECM that has been shown to facilitate tumor growth, cancer cell migration, and metastatic invasion^3^, and induce interstitial hypertension. This hypertension or excessive tissue pressure directly results in widespread vascular collapse^2, 4, 5^ and reduced molecular uptake^6^. In this study, this spatial relationship was investigated at the local/regional level, for key stromal components hyaluronic acid (HA) and collagen. The study quantified elevated interstitial hypertension and the subsequent influence on vascular patency and molecular delivery to PDAC, analyzing the spatial relationship of collagen relative to HA and vessel patency throughout many points in each tumor.

The measurable mechanical properties of the tissue, including viscoelasticity and tumor stiffness, are strongly affected by the enhanced deposition of collagen and hyaluronic acid within the ECM^7^. Growth-induced solid stress (SS) is a prominent measurable feature of tumor stiffness, and is defined as the stress applied by the migration and proliferation of cancer cells that is subsequently stored by structural elements within tissue^8^. This SS is essentially a mechanical pressure from solid elements within the tissue, and excludes any fluid pressure. Hyaluronic acid provides resistance to compression in tumors due to both electromechanical repulsion of the negatively-charged glycosaminoglycan^7^ and propensity to imbibe interstitial fluid. This combination of both electromechanical repulsion and osmotic pressure results in swelling of the tumor matrix^9^, which is elastically confined by collagen fibers, thereby transmitting stress outwards to squeeze lower pressure components, inducing blood and lymphatic vessel collapse^10^. These nonfunctional lymphatics then limit fluid and plasma macromolecules from draining out of the tumor interstitium, leading to free fluid accumulation and elevated interstitial fluid pressure (IFP)^11^. As IFP increases to the value of microvascular pressure (MVP), convective transport of macromolecules out of the microvasculature desists, leaving diffusion as the primary mechanism for delivery from vessels to tissue interstitium in pancreatic cancer. This large cascade of stiffness and pressure driven tissue features leads to the fact that large parts of the PDAC tumor can be impenetrable to delivery of systemic therapeutics.

The micro-piezoelectric pressure transducer used in this study has been referred to as a gold standard in measuring tissue pressure in both positive and negative pressure regimes^12^. The inherent nature of a piezoelectric device is that it measures all applied stress on the active element lateral to the direction of insertion, thereby integrating together total tissue pressures (TTP), which are thought to be a combination of both growth induced solid stress (SS) and IFP^13, 14^. This has been illustrated in numerous studies^8, 13, 15, 16^, where the piezoelectric sensor has measured pressure values considerably larger than the wick-in-needle technique^17^, which is known to measure IFP. A previous study^14^ developed a modified attachment to the piezoelectric transducer to separate and quantify TTP and IFP in the same tumor location, thereby indirectly quantifying SS. That earlier study showed a direct correlation between TTP, measured predominately as SS, with collagen area fraction. However, the intrinsic heterogeneity in vascular patency and molecular uptake proved that a single localized, point-based measurement of TTP was insufficient to draw any conclusions on potential correlation with the entire tumor, especially when the histology information was not directly sampled from the immediate areas of the pressure measurements, as has been common in previous studies by many researchers. This motivated the current study, hypothesizing that multiple locations of measurements within tumors are needed to understand the extreme heterogeneity which could be present, and that this could only be fully uncovered with co-located histology analysis of the immediate areas around the pressure measurements.

In this study, two human pancreatic tumor lines, AsPC-1 and BxPC-3, were studied for the spatially correlated collagen and HA, vascular patency and *in vivo* drug delivery of verteporfin, as related to total tissue pressure (TTP), solid stress (SS) and interstitial fluid pressure (IFP). Localized, analysis was conducted to determine the interplay between these tumor parameters and test the above hypothesis about how micro-regional pressure and collagen values are﻿ in their impact.

## Materials and Methods

### Animals and Tumor Models

All animal procedures were conducted under the protocol approved by the Dartmouth Institutional Animal Care and Use Committee (IACUC), and all procedures directly followed approved procedures and guidelines required in the approved protocol and regulations governing these procedures. Two human PDAC xenograft tumor lines, AsPC-1 (ATCC, Cat#CRL-1682) and BxPC-3 (ATCC, Cat#CRL-1687), were used with 30 female athymic nude rats, between 6 and 8 weeks of age, being implanted via injection of 1 × 10^6^ cells, in Matrigel (BD Biosciences, San Jose, California), subcutaneously into the right flank and orthotopically directly into the pancreas. Both subcutaneous and orthototopic locations were incorporated into this study, because tumor growth, metastatic potential, and efficacy of systemic treatments are influenced by the host-tumor microenvironment^18^. The tumors were allowed to grow for a period of 4 to 6 weeks for AsPC-1 tumors and 6 to 10 weeks for BxPC-3 tumors, or until reaching an average diameter of 10 to 15 mm for orthotopically grown tumors and 5 to 8 mm for subcutaneously grown tumor.

### Total Tissue Pressure (TTP) and Interstitial Fluid Pressure (IFP) Measurements

A Millar Mikro-tip piezoelectric pressure catheter (model SPR-671, 0.47 mm diameter) was used in this study to measure total tissue pressure, TTP. A modified attachments, illustrated previously^14^, was implemented to quantify IFP and TTP within the same tumor location, thereby indirectly quantifying SS. To place the pressure catheter into tumor tissue, a 23-gauge needle was first introduced into the tissue and the pressure catheter was quickly placed into the track created by the needle. The catheter was removed and reinserted several times to avoid adherence to the surrounding tissue. The active pressure sensor was approximately located 3 to 5 mm below the tumor surface.

Three tumor locations were measured sequentially for each tumor; a single location for IFP and all three locations were measured for TTP. The tumor locations were separated by approximately 4 mm and were located along the same horizontal plane, illustrated in *S.I Materials and Methods*. India ink was incorporated to mark the locations of each pressure measurement location. Data were collected for a minimum of 10 minutes for each tumor location or until a constant pressure value was observed.

### Image Quantification

#### Collagen and Hyaluronic Acid Area Fraction

**Figure 1:**
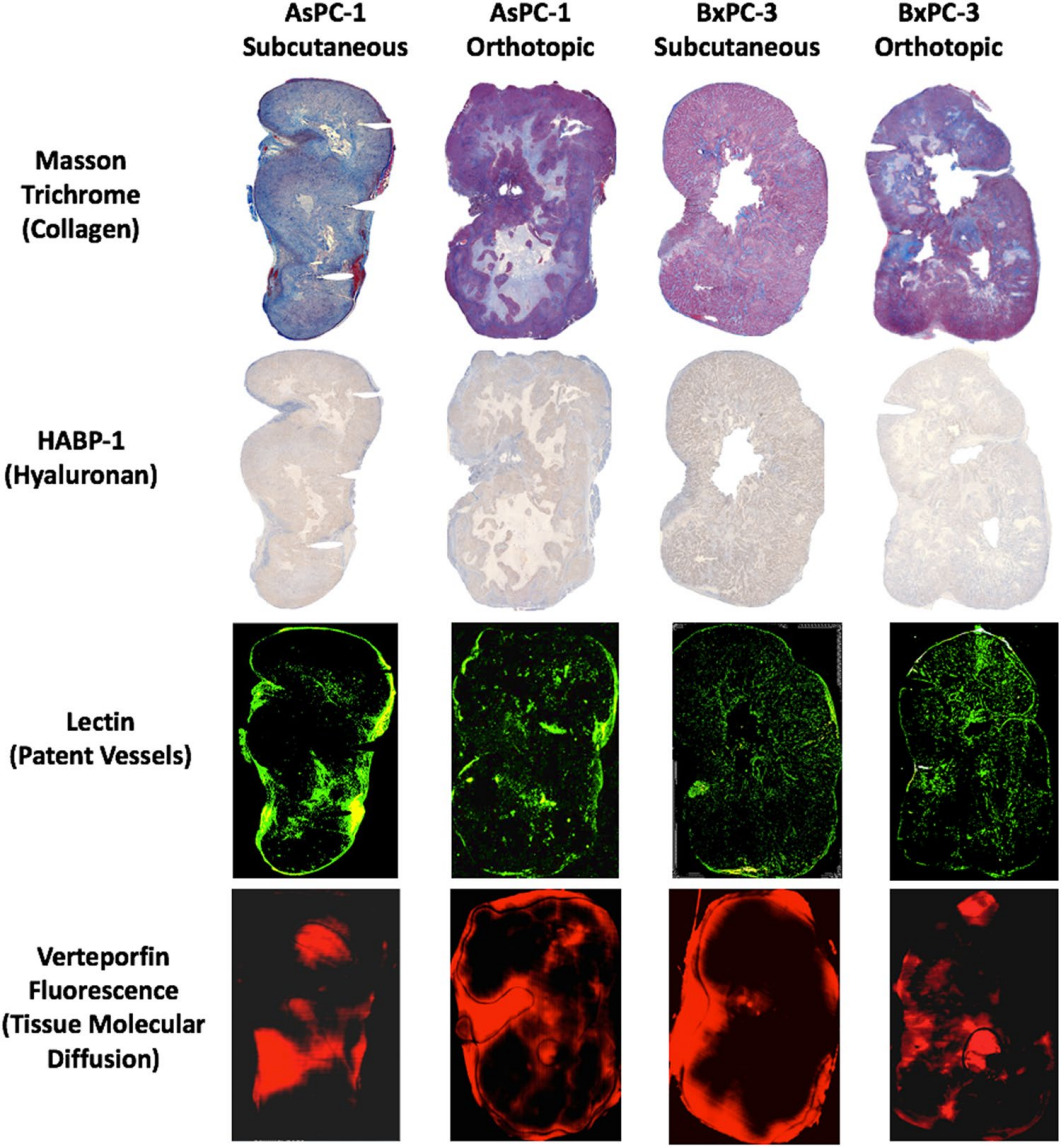
Two human pancreatic tumor lines, AsPC-1 and BxPC-3, were implanted in both subcutaneous and orthotopic locations. Stromal components, i.e. collagen and hyaluronan, patent blood vessels and tissue molecular uptake, were analyzed across an entire histological tumor slice.

**Figure 2:**
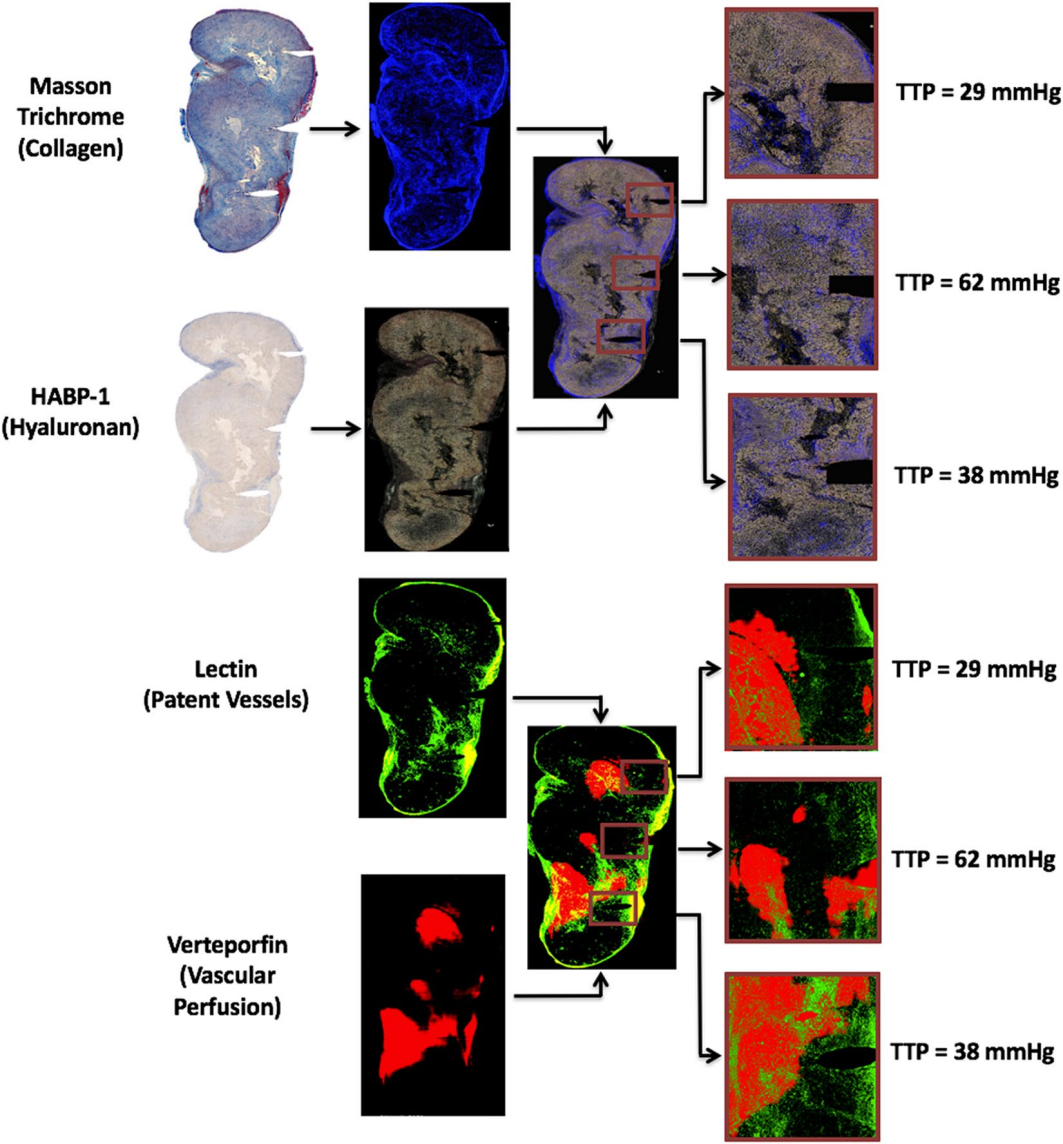
The experimental procedure for localized tumor analysis of TTP is shown, Stromal components (collagen and hyaluronic acid), vascular patency, and verteporfin perfusion. *In-vivo* measurements of TTP were taken at three planar locations within the pancreatic tumor. The tumor was excised and *ex-vivo* analysis was conducted to correlate local measurements of TTP with surrounding collagen area fraction, hyaluronic acid area fraction, patent vessel area fraction, and mean fluorescence intensity of verteporfin, in the exact same regionally defined locations.

After pressure acquisitions, both subcutaneous and orthotopic tumors were excised. A 25-gauge needle was placed into each pressure measurement location track to maintain the opening location, and then the tumor was formalin fixed. The tumor was cut in half, fixed and paraffin embedded for thin sectioning (4 μm). Serial sections of each tumor were stained with one of H&E, Masson’s Trichrome (MT) stain, and Hyaluronic Acid Binding Protein (HABP-1), as well as the vascular and tissue perfusion markers described later. Sections were then imaged with 10 × magnification using PerkinElmer Vectra3 slide scanner, providing high resolution digital images of each stain for the entire tumor section, as shown in Figs 1 and 2.

Tumor sections stained with MT and HABP-1 were analyzed separately with color segmentation to extract collagen and HA fraction, respectively. The MT images were converted from RGB to HSV (Hue-Saturation-Value) color space, providing an efficient method to separate color intensity. For collagen, the threshold hue, saturation, and value numbers used were 0.6–0.7, 0.7–1, 1, respectively. This segmentation is described further in *SI Materials and Methods*. HABP-1 was analyzed by extracting brown pixels within the RGB image. The algorithm achieved both white background subtraction and located regions where the red image plane pixel intensity is 2x greater than the green and blue image plane pixel intensities. The segmented color images of collagen and hyaluronic acid were coregistered (cpselect function in MATLAB) with 5 to 8 matching surface features. A linear conformal rigid transform (tform in MATLAB) was generated using these input image locations to align the tumor boundary of each related image. An illustration of color segmentation and coregistration is shown in Fig. 2.

**Figure 3:**
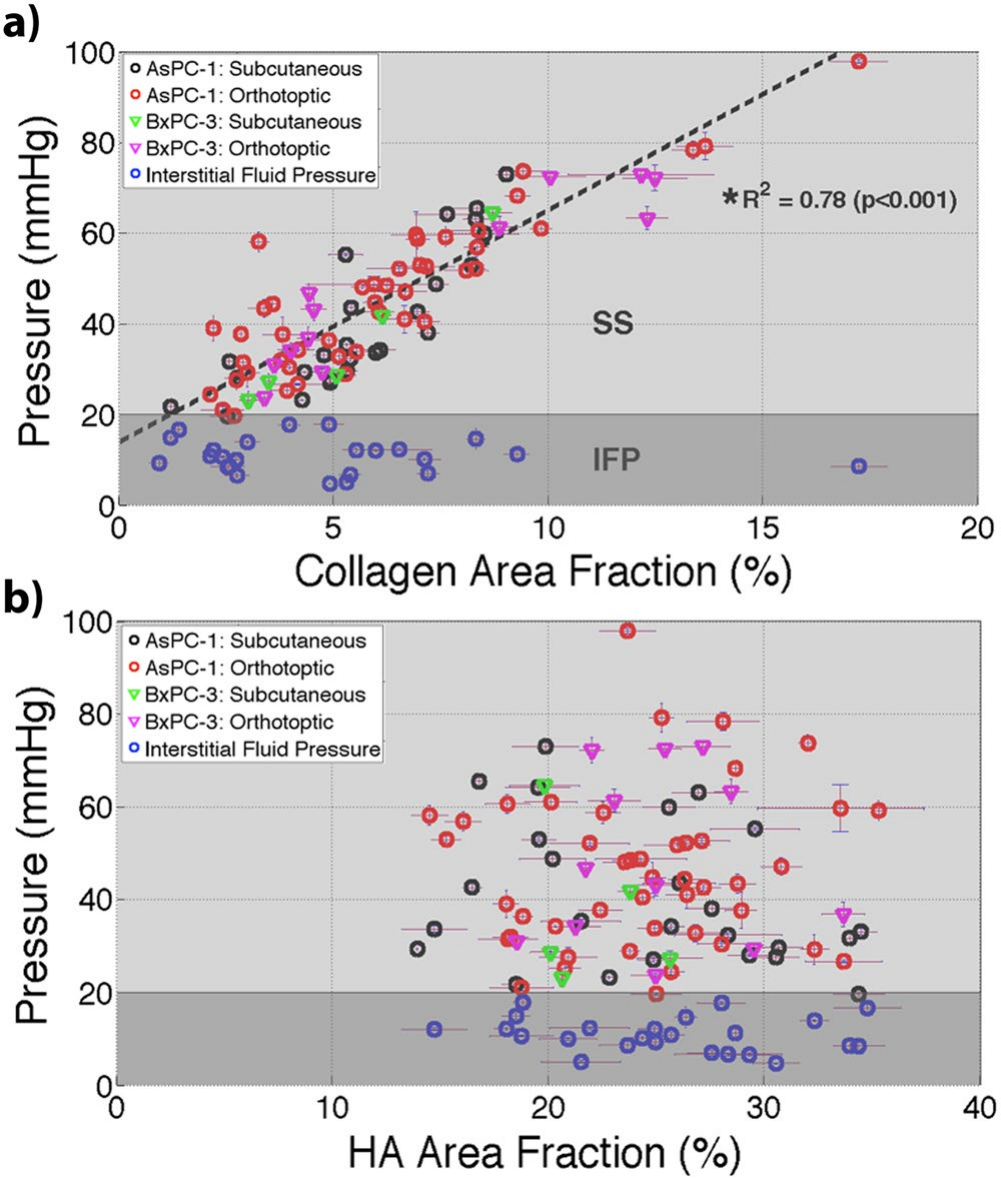
Pressures measured in all locations of both AsPC-1 and BxPC-3 tumors. TTP *in vivo* was plotted with respect to *ex vivo* measures of (**a**) collagen area fraction (%) and (**b**) hyaluronic area (HA) fraction (%). A strong correlation was observed between collagen area fraction and TTP using a linear regression fit (R^2^ = 0.78; *p < 0.001). No significant correlation existed between TTP and hyaluronic acid area fraction.

A region of interest (ROI) was defined at the location of each pressure measurement, identified either by physical deformation, as illustrated in Fig. 2, or by India ink location on the surface of the tumor. The ROI was selected to be a square with side lengths of 0.5, 1, 1.5, and 2 mm, corresponding to 1, 2, 3, and 4 times the diameter of the piezoelectric pressure sensor; the area within the needle track was ignored within this analysis. Collagen area fraction (%) and HA fraction (%) were quantified by calculating the number of pixels from MT and HABP-1 stained images, respectively, normalized to the total number of pixels in each ROI. The mean collagen area fraction (%) and HA area fraction (%) for each tumor are displayed in Fig. 3a and b.

#### Texture Analysis

Intratumoral heterogeneity is a recognized feature of malignancy often associated with higher tumor grades and poor prognosis^19, 20^. Texture analysis was used to evaluate collagen heterogeneity in localized ROI’s through quantification of entropy, uniformity, and fractal dimension. Entropy is a measure of irregularity in the distribution of collagen within tissue and quantified using histogram analysis by the equation: ${\rm{Entropy}}=-{\sum }_{{\rm{I}}=1}^{{\rm{N}}}({\rm{P}}({\rm{V}})){\mathrm{log}}_{2}({\rm{P}}({\rm{V}}))\,$, where P(V) represents the intensity occurrence probability and V represents the area fraction of collagen^21^. Uniformity is a measure of how close the image is to a uniform distribution and is quantified by the equation: ${\rm{Uniformity}}={\sum }_{{\rm{I}}=1}^{{\rm{N}}}{[{\rm{P}}({\rm{V}})]}^{2}\,$. Fractal dimension is a quantitative method to define complexity of collagen distribution within tumors. The box-counting method^22^ was applied to each ROI and the fractal dimension was computed by identifying ${\rm{D}}=\frac{\mathrm{log}({\rm{N}})}{\mathrm{log}(1/{\rm{e}})}$, where N represented the number of boxes containing collagen and e represents the side length of each box. The results for each of these were compared for association with co-localized measurements of TTP.

#### Patent Vessel Area Fraction

**Figure 4:**
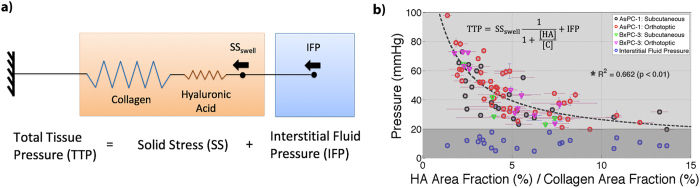
Confinement of Hyaluronic Acid by collagen directly increases SS, thereby contributing to elevated TTP. A simple model was illustrated in (**a**) representing TTP as a combination of mean IFP measured in this study and SS, which is represented by a series of springs, collagen (C) and hyaluronic acid (HA), and a constant swelling stress (SS_swell_). The data from this study (**b**) was fit to the model (inset) with SS_swell_ fit to 170 mmHg. [HA] and [C] represent the area fractions for both HA and C, respectively.

Vascular patency was measured using an injected fluorescent stain, Lectin (Vector Laboratories, Cat# FL-1211), administered i.v. at a concentration of 1 mg/kg, one minute prior to sacrifice. The tumor was excised, paraffin embedded, and thinly sectioned (4 μm) as previously described. The serial sections adjacent to the MT & HABP-1 images were used for this, and were digitized at 10 × magnification using the PerkinElmer Vectra3 fluorescence slide scanner, using the FITC filter setting. The fluorescence image of lectin was coregistered with MT and HABP-1 images, to ensure reasonable spatial correlation. Patent vessel area fraction (%) was quantified by calculating the number of pixels stained with lectin compared with the total number of pixels in each ROI, shown in Fig. 2. The mean patent vascular area fraction (%) from four ROI’s was reported in Fig. 4a.

#### Verteporfin Uptake Measured Through Fluorescence Intensity

Verteporfin (Sigma-Aldrich) is a photosensitizer used in photodynamic therapy (MW = 719, empirical formula C_41_H_42_N_4_O_8_), and was used here as a fluorescent contrast agent to study small molecule perfusion within the tumors. Verteporfin was administered i.v. at a concentration of 1 mg/kg and allowed to circulate for one hour. After the tumors were excised and cut in half, each slide was imaged for fluorescence at 690 nm on a flatbed scanner (GE Typhoon 9410). The fluorescence images of verteporfin were also coregistered with MT and HABP-1 stained images, to ensure reasonable spatial correlation. Verteporfin uptake measured through this fluorescence intensity (A.U) was quantified by calculating the mean intensity in each ROI, shown in Fig. 2. The mean fluorescence intensity from four ROI’s was reported for both subcutaneous and orthotopic tumor locations in Fig. 4b.

### Collagenase Administration to Actively Degrade Collagen *In Vivo*

Active treatment to enzymatically degrade collagen was examined in a sub-study by application of three 50 μL injections of Collagenase-D (Sigma Aldrich, Cat. No. 10103578001) in each tumor used, at a concentration of 0.5 mg/mL, concentration was provided by Voutouri *et al*.^5^. Measurements of TTP were conducted at two locations one minute prior to administration of collagenase and 1 hour post-administration. The resulting change in TTP pre- and post-administration of collagenase was studied. Verteporfin was administrated one hour post-administration of collagenase, by i.v. (concentration of 1 mg/kg) and allowed to circulate for one hour. The tumor was excised and the fluorescence intensity of verteporfin was imaged using the flatbed scanner as before, and results were compared with control tumors with no administration of collagenase.

### Statistical Analysis

Linear regression was used to test for correlation between parameters as measured from the same locations within the tumor tissues, such as pressure and collagen, hyaluronic acid, and vessel density. The ratio of hyaluronic acid to collagen area fraction was fit using a simple Hookean model, defined below, where the fit parameter, SS_swell_, was optimized to achieve the largest coefficient of determination, R^2^. Several data sets were used to test for differences in their mean values. In the cases where the sample sizes were not equivalent, a Welch t-test was used to compare data sets with unequal sample sizes and variance. This was applied to comparing the residuals between untreated tumor with collagenase applied tumor data sets.

## Results

### Confinement of Hyaluronic Acid by Collagen Increases Total Tissue Pressure in Pancreatic Tumors

One hypothesis going into this work was that increased collagen content, measured through collagen area fraction, would directly correlate with TTP, across tumor sites, tumor types and different intratumor locations. Additionally, increased confinement of hyaluronic acid by collagen, measured by a ratio of hyaluronic acid area fraction (%) to collagen area fraction (%), would translate to elevated TTP, as collagen will constrict volumetric increase of hyaluronic acid imparting internal stress to the microenvironment.

The results of this study illustrated a strong correlation between TTP and collagen area fraction (%), Fig. 3a, across two pancreatic xenograft lines, AsPC-1 and BxPC-3, and two tumor locations, subcutaneous and orthotopic. Mean values for each tumor were fit using linear regression with R^2^ = 0.78 (p < 0.001). The p-value was calculated under the null hypothesis of no correlation between TTP and collagen area fraction. An approximate microvascular pressure of 20 mmHg^23^ was marked to illustrate the data range differences between interstitial fluid pressure (IFP) from solid stress (SS). The mean values of TTP and IFP for all tumors analyzed were 44.3 mmHg and 10.9 mmHg, respectively, indicating SS easily dominates TTP within highly stromal tissues such as pancreatic tumors. Similar mean values measured have been reported in other studies involving pancreatic human xenograft tumors^14, 16^.

No apparent correlation existed between TTP and hyaluronic acid area fraction (%), suggesting TTP was not directly influenced by the physical area hyaluronic acid occupied within the tumor interstitium, Fig. 3b. Voutouri *et al*.^5^ showed a similar result whilst comparing external swelling stress to hyaluronic acid area fraction.

A proposed model for the intrinsic interactions of the confinement of hyaluronic acid by collagen is shown in Fig. 4a, derived from the standard solid model for viscoelastic tissue^24^, described in *S.I Materials and Methods*. Solid Stress (SS) and Interstitial Fluid Pressure (IFP) are represented as independent systems, where the summation of both equates to total tissue pressure (TTP). IFP is defined by the mean IFP measured using the modified piezoelectric transducer in each tumor location. SS is defined by a series of springs representing collagen and hyaluronic acid, and a constant swelling stress, SS_swell_. By solving the simple elastic model, TTP is defined by: ${\rm{TTP}}={{\rm{SS}}}_{{\rm{swell}}}\frac{1}{1+\,\frac{[{\rm{HA}}]}{[{\rm{C}}]}}+{\rm{IFP}}$, where [HA] and [C] represent hyaluronic acid and collagen area fractions, respectively, and SS_swell_ was a fitting parameter used to maximize the coefficient of determination, R^2^.

A strong correlation existed between TTP and the ratio of HA to collagen area fractions, Fig. 4b. The optimal fitting parameter SS_swell_ was determined to be 170 mmHg resulting in an R^2^ = 0.66 and p-value < 0.01. The mean IFP for all tumors analyzed was 10.9 mmHg. This result suggests that as collagen becomes more dense within the tissue interstitium, advanced confinement of hyaluronic acid results in elevated TTP.

### Increased Irregularity and Complexity of Collagen Distribution Correlates with Elevated TTP

**Figure 5:**
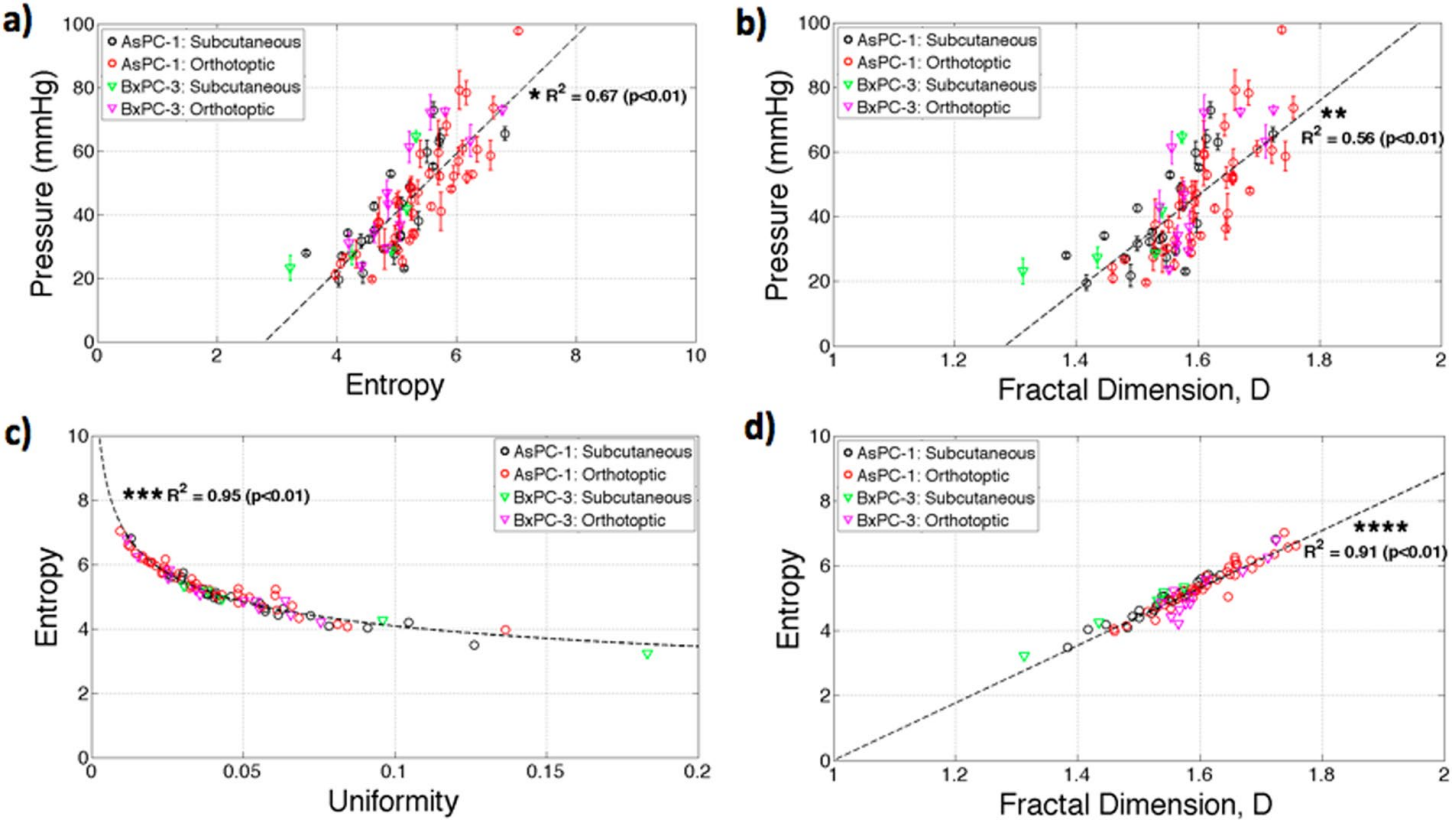
Texture analysis of collagen distribution was conducted on the tumor images. Entropy (**a**) and fractal dimensions (**b**) were compared to TTP in localized ROI’s resulting in R^2^ values of *0.67 and **0.56, respectively. Entropy was correlated with both uniformity (**c**) (***R^2^ = 0.95; fit: x^−0.25^) and fractal dimension (****R^2^ = 0.91) indicating that more heterogeneous distribution of collagen directly results in elevated TTP.

The results presented in Fig. 5a and b indicate both entropy and fractal dimension, D, correlate to TTP withR^2^ values 0.67 and 0.56, respectively. These texture parameters suggest that elevated collagen area fractions in tumors have increased irregularity and complexity in distribution patterns, thereby resulting in increased TTP and potentially reduced molecular uptake. Additionally, Fig. 5c and d indicate strong correlation between entropy and uniformity, R^2^ = 0.95 (power law fit: x^−0.25^) and correlation between entropy and fractal dimension, R^2^ = 0.91, indicating that larger values of TTP occurs with more heterogeneous distribution of collagen within tumors.

### Localized Measurement of TTP Directly Correlates to Reduced Vascular Patency and Drug Delivery

**Figure 6:**
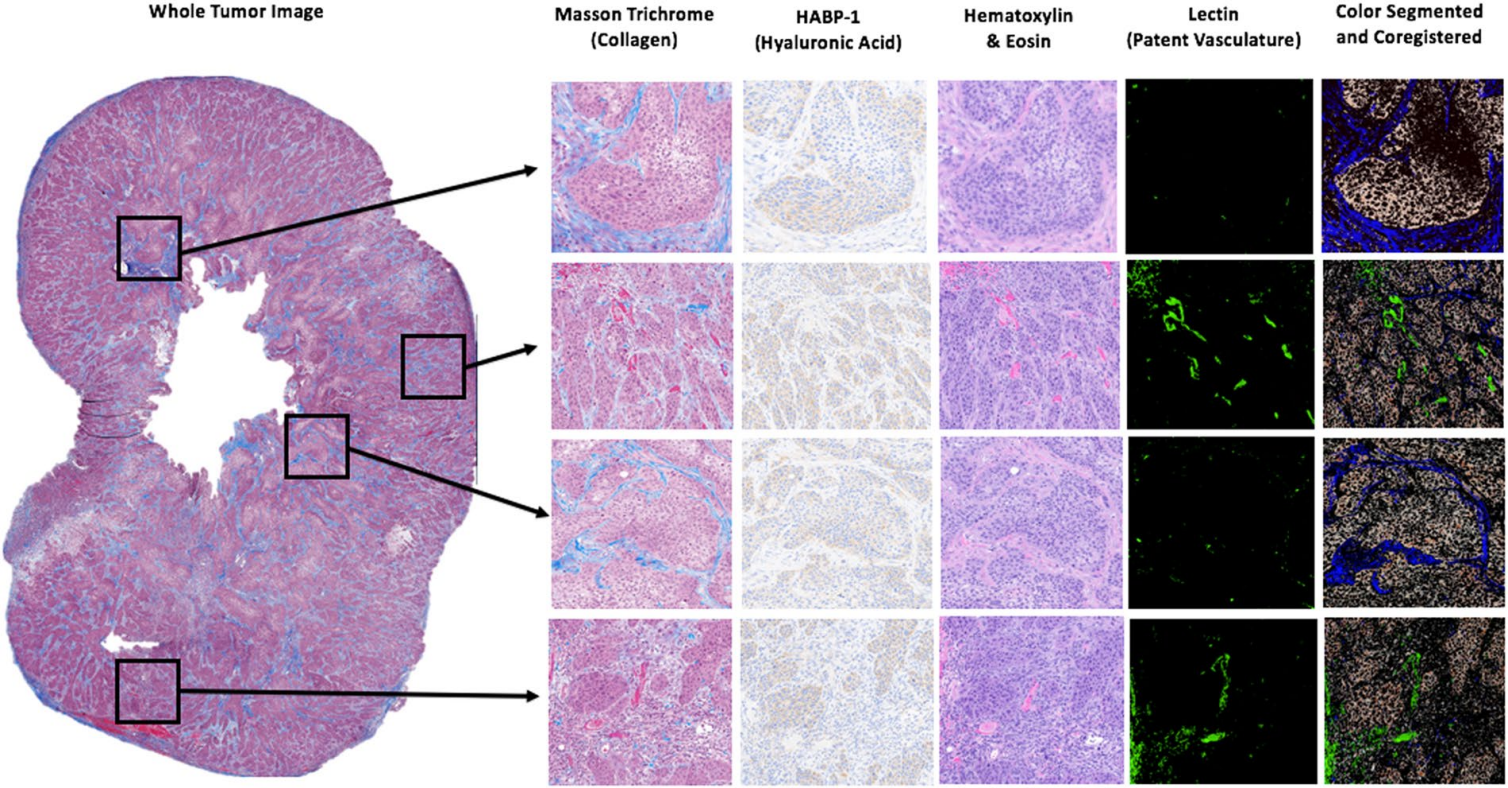
Whole tumor images were analyzed locally for H&E, MT, HABP-1, and Lectin. Rows 1 and 3 indicate regions of collagen encapsulating hyaluronic acid, thereby resulting in elevated TTP and minimal functional vasculature. Rows 2 and 4 indicate regions with limited collagen and active vasculature.

It was hypothesized that increased deposition of collagen leads to a buildup in TTP, thereby resulting in vascular collapse and reduced molecular uptake, so analysis of this phenomenon was carried out and illustrated in Fig. 6. As shown, row 1 and 3 illustrate regions with increased confinement of HA by collagen, resulting in virtually no functional vasculature. Rows 2 and 4 indicate areas where HA is minimally confined by collagen, thereby resulting in active blood flow. These observations help visualize the nature of the correlation between TTP, vascular patency, and verteporfin uptake within pancreatic tumors.

**Figure 7:**
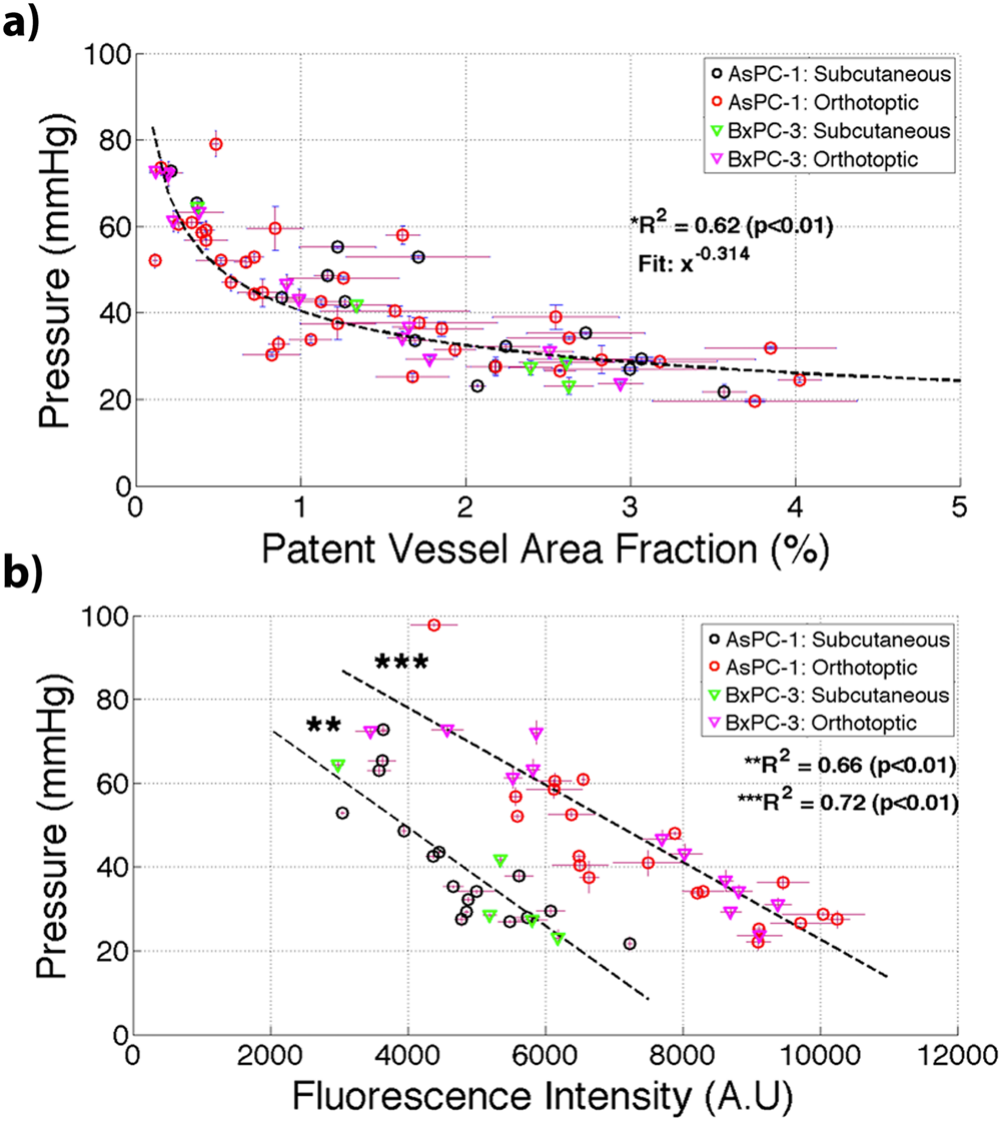
TTP was compared to (**a**) patent vessel area fraction (%) and (**b**) verteporfin uptake measured through fluorescence intensity (A.U) analyzed *ex-vivo*. A statistically significant correlation existed between TTP and patent vessel area fraction (%) with a power law dependence relationship (x^−0.31^, *R^2^ = 0.62, p < 0.01). A statistically significant correlation existed between TTP and verteporfin fluorescence for both subcutaneous (**R^2^ = 0.66, p < 0.01) and orthotopic (***R^2^ = 0.72, p < 0.01) tumors.

These results presented in Fig. 7 show a strong inverse power correlation between TTP and patent vessel area fraction, with power law dependence of x^−0.31^. A linear regression fit was performed on the natural logarithm of TTP and patent vessel area fraction, yielding an R^2^ = 0.62 and p-value < 0.01. A correlation between patent vascular area and TTP measurement has been shown in other studies^2, 14^.

The mean values of verteporfin uptake were acquired for subcutaneous and orthotopic locations and compared with localized measurements of TTP for each tumor. The results showed a statistically significant decrease in verteporfin uptake in orthotopic tumors (R^2^ = 0.72; p < 0.01) and statistically significant decrease in subcutaneous tumors (R^2^ = 0.66; p > 0.01). Verteporfin uptake was elevated in orthotopic tumors as compared with subcutaneous tumors, similar to previous findings with the AsPC-1 tumor line^14^.

### Enzymatically Degrading Intratumoral Collagen Reduces TTP and Increases Drug Delivery

**Figure 8:**
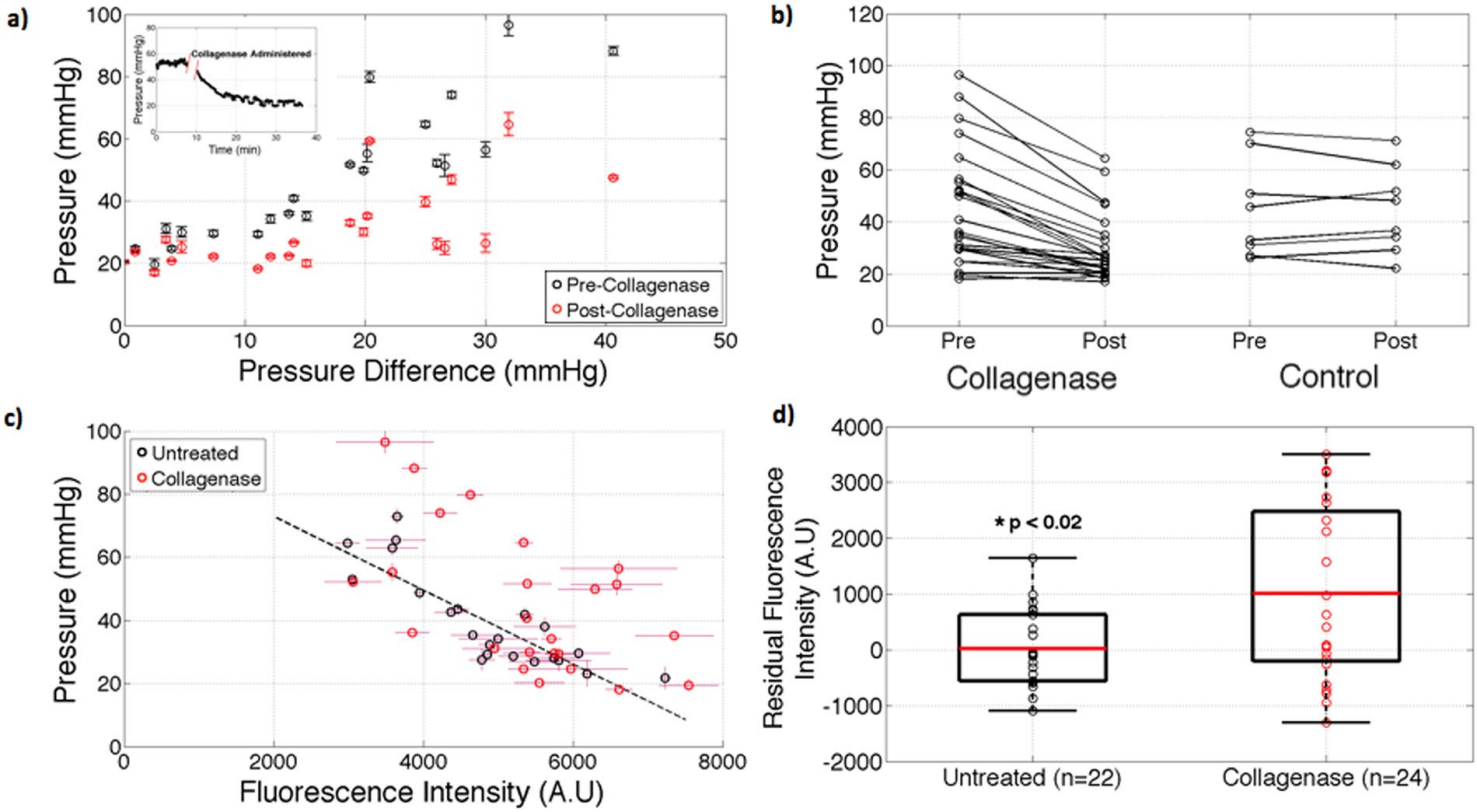
Collagen was enzymatically degraded in AsPC-1 tumors by intratumor injection of Collagenase and pressures pre- and post-administration, were measured, showing a decreased in all tumors studied (**a**), with a mean TPP reduction of 30%, as compared to the control (change of 0.8%). The individual data is shown before and after as a function of pressure difference (**a**) and in pre vs. post line plots (**b**). Verteporfin fluorescence post collagenase was also compared to fluorescence data presented for untreated tumors (**c**). Residuals between untreated and collagenase treated tumors regression fits were compared as individual data points (**d**) illustrating a statistically significant increase in verteporfin uptake in collagenase treated tumors.

It was hypothesized that alleviating the tensile stress posed by collagen within the tumor would allow internal relaxation of hyaluronic acid, thereby resulting in a net reduction in TTP, decompression of vasculature, and an increase in verteporfin uptake. Our results in Fig. 8a showed TTP significantly decreased in tumors treated with collagenase, mean reduction of approximately 30%. Similar percent reductions in pressure was reported by Gade *et al*.^25^ in HT29 xenografts treated with collagenase.

These results in Fig. 8b indicated that administration of collagenase significantly increased verteporfin uptake. Residuals were measured by comparing untreated tumor fluorescence intensity and collagenase treated tumor fluorescence intensity, against the predicted linear regression fit. A statistically significant increase in verteporfin uptake was determined using a Welch t-test (p < 0.02). These results suggest that transient SS relief can increase molecular uptake within the tumor in an acute delivery such as this, leading to more uniform distribution of molecules within the tumor interstitium.

## Discussion

The goal of this study was to investigate the heterogeneity of PDAC in collagen and hyaluronic acid distribution and the inherent role these parameters have on SS in pancreatic tumors. There is wide acceptance amongst researchers that increased deposition of hyaluronic acid and collagen directly leads to elevated interstitial pressure^5, 6, 11, 14–16^; however, the level of influence on local pressures and stresses, and the interplay between these matrix components has received only speculation and limited data measurements *in vivo* ^5, 6, 16^ and the sampling of multiple points within tumors remains largely unexplored. DuFort *et al*.^16^ suggested that hyaluronic acid has a dominant role in interstitial pressure measured with a piezoelectric pressure catheter, as both hydrostatic and oncotic pressures result in swelling of hyaluronic acid, thereby causing vascular collapse. They suggested the pressure measured using a piezoelectric pressure catheter was a balance between hyaluronic swelling stress and collagen contraction. This assertion agreed with asymptotic relationships between hyaluronic acid concentration and interstitial pressure measured across several pancreatic tumor lines, implying a balance between these two forces; however, evidence related to collagen concentration within these tumors was not as fully explored.

The results presented in Fig. 3 show the local relationships between measured TTP, hyaluronic area fraction, and collagen area fraction. Through this localized analysis, a strong direct correlation was seen between TTP and collagen area fraction (Fig. 3a) and yet no apparent correlation was seen between TTP and hyaluronic acid (Fig. 3b). These findings indicate that collagen plays the more dominant role in inducing TTP, as predominantly from collagen SS, in PDAC. Still the presence of HA is known to be important in amplifying the SS by providing the swelling force which translates the collagen stress outwards to the epithelial and endothelial components of the tissue.

**Figure 9:**
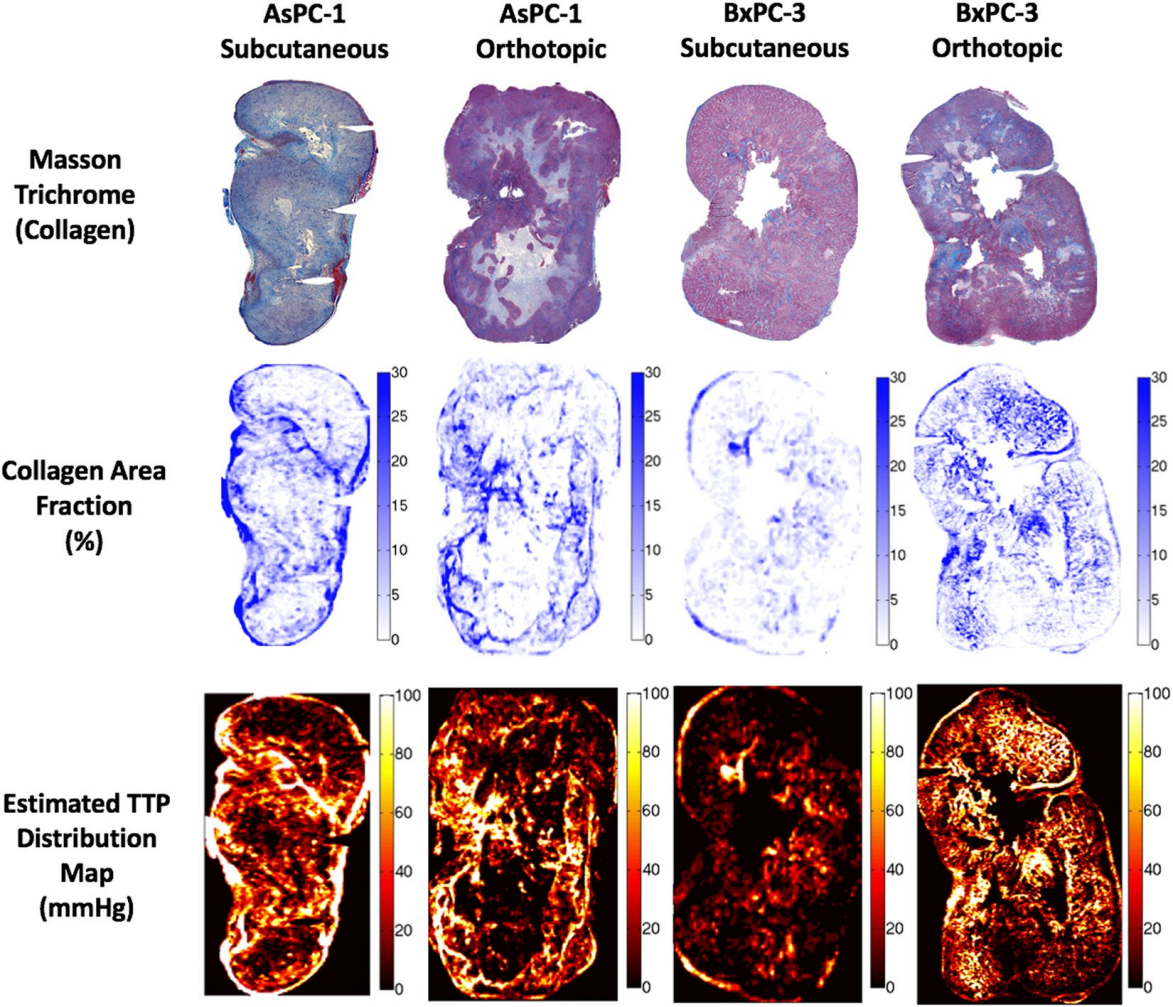
Estimation of whole tumor TTP based upon collagen distributions. Each tumor was divided into 100 micron regions and collagen area fraction was computed. The results from Fig. 3a where applied to model what the TTP distributions should look like within an entire tumor slice, illustrating the extreme level of microscopic pressure heterogeneity which this study indicates should exist within the tumor.

The bulk stiffness of tissue increases with increased collagen content^26^, thereby further impeding mechanical deformation, resulting in stored mechanical stress within the region tissue around the collagen build up. However, the TTP area affected by the stroma would appear to be quite localized to several hundred microns of tissue nearest the collagen fibers. If true, this strong correlation between collagen area fraction and TTP allows for model projections of TTP within an entire tumor from knowledge of the collagen distribution, as illustrated in Fig. 9. Knowledge of whole tumor TTP is essential in further understanding internal stress within tumors and spatially heterogeneity, which clearly exists and is an impediment to molecular uptake.

An important analysis developed in this study was the proposed elastic model to describe intrinsic interaction between hyaluronic acid swelling and collagen. A constant swelling stress was applied to a series of springs, representing collagen and hyaluronic acid (Fig. 4a), and this model provided an excellent fit to experimental data, as shown in Fig. 4b. The results indicated that elevated TTP was a direct result of collagen confinement of hyaluronic acid, thereby suggesting that collagen distribution serves as a pathological indicator for high pressure, reduced vascular patency and molecular uptake. A SS_swell_ of 170 mmHg was reported as an approximate hyaluronic acid swelling stress, induced by both electromechanical repulsion and osmotic equilibrium, to compress collagen fibers and impart localized elevated pressure within the tumor tissue. Lai *et al*.^27^ showed similar results *in-vitro* collagen-hyaluronic acid hydrogels where volumetric swelling of hyaluronic acid was restricted in the presence of collagen, resulting in increased residual stress. Additionally, similar results were shown by a direct correlation between tensile modulus to the ratio of collagen/hyaluronic acid content in articular cartilage^28^.

The significance of dividing TTP into a constant IFP pressure source and an elastic model for SS was indirectly justified by DuFort *et al*.^16^. In that work, DuFort suggested that pressure within pancreatic tumors is divided into two phases, a rapidly increasing, saturable component representing hyaluronic acid swelling and contractile confinement by collagen, and a linearly rising, unsaturable component representing fluid pressure. In their model, the saturating component was fit according to the following equation, $\frac{[HA]}{[HA]+K}$, where K represented a fitting parameter to describe the contractile stress applied by collagen; however, collagen was not quantified in that study. This study illustrates that this fit matches the derivation of a simple elastic model where [HA] and K represent the area fractions of hyaluronic acid and collagen, respectively. This model can accurately predict TTP within pancreatic tumors, further justifying the notion that SS is a result of the intrinsic interactions between hyaluronic acid swelling and collagen distribution.

The important implications of SS have led to new developments to quantify this parameter directly through *ex-vivo* and *in-vivo* means, and allowing an interpretation of the level of spatial heterogeneity which likely exists. Stylianopoulos *et al*.^8^ demonstrated bulk estimation of SS by cutting excised animal tumors and measuring stress relaxation as the extent of tumor opening normalized by the diameter of the tumor. Voutouri *et al*.^5^ quantified bulk estimation of swelling stress in tumors with enhanced desmoplasia by confined compression using a mechanical testing system. High-resolution ultrasonography has been used to quantify 2D mapping of solid stress induced deformation in tissue^29^, and this method was expanded to monitoring deformation in biopsy cores, providing regional quantification of solid stress and extending to *in-vivo* clinical application. Additional ultrasonography measurement techniques to quantify tissue stiffness include harmonic motion imaging (HMI)^30^, model-based ultrasonic elastography imaging^31^, and ultrasound shear-wave elastography^32^. These techniques either quantify bulk estimations of stress or regional elasticity of tissue, however, the essential parallel between internal stress and pathological features are often ignored in macroscopic type studies. However, the work here illustrates how the originating mesoscopic collagen matrix distribution and spatial scale can dictate the level and size distribution of pressure heterogeneity which exists.

Textural analysis is used determine image spatial heterogeneity, in x-ray, ultrasound, computed tomography (CT), and magnetic resonance imaging (MRI)^19^, and can serve as a predictor of tumor grade and patient survival rate^20^. Figure 5 illustrated that TTP directly correlates with increased entropy, reduced uniformity, and increased fractal dimension in collagen distributions in pancreatic tumors. These results suggest that elevated collagen density with tumors possess increase spatial irregularity and complexity, compared with tumors with lower TTP. This pathological feature could serve as a non-invasive indication of the aggressive deposition of collagen within tumors and can indirectly indicate regions with reduced vascular patency and reduce molecular uptake. Further, as the complexity of a tumor increases, so does textural complexity, and the likelihood of systematic therapies being ineffective. This microscopic analysis of pressure is one further link between the phenomenological observations of decreasing response with increasing complexity, and perhaps providing some explanation of how heterogeneity of pressure in the tumor can be a causal feature of the lack of response.

Regional quantification of SS, measured through TTP, provided direct insight into vascular patency and molecular distribution with pancreatic tumors. The results in Fig. 7 illustrate a power law dependence of increased TTP in respect to decreased patent vessel area fraction (a) and a direct correlation between elevated TTP and reduction in verteporfin uptake (b), measured by fluorescent intensity; these results coincide with the similar findings in other studies^2, 4, 6, 14^. The inherent heterogeneity of TTP within a single tumor, as illustrated in Fig. 5, illuminates a significant impediment to systemically delivered therapies, as patent vessels and verteporfin uptake will be localized in regions with reduced solid stress, producing local hotspots of molecular uptake. Verteporfin transport is primarily diffusion-weighted, indicating increased SS will prohibit molecular transport due to limited functional vasculature, as well as limiting intratumoral diffusion due to the diffusion coefficient increasing with hyaluronic acid and collagen content^33^.

Degradation of collagen within the ECM of pancreatic tumor should then reduce SS, thereby decompressing vasculature and increase verteporfin uptake. Unfortunately, these degradation mechanisms are often transient and hard to sustain without systemic morbidity or toxicity effects. Nonetheless, the study completed here, while acute, helped verify that a reduction in TTP would correspond to significant increased potential for vascular molecular delivery. These findings match with several studies^4, 5, 25^ indicating that elimination of tensile stress applied by collagen, allows intratumoral swelling of hyaluronic acid and net SS relaxation. Reduced SS will in turn reopen vascular pathways for molecules to enter the tumor interstitium. Alternatively, relieving the force applied to the collagen matrix by degrading hyaluronic acid conceivably could reduce TTP within tumors as well. Provenzano *et al*.^2^ demonstrated this by administering PEGPH20 to autochthondous pancreatic tumors and measuring a 4-fold reduction in TTP and a ~70% increase in discernable vessel diameter. Due to the extensive research conducted in enzymatically degrading hyaluronic acid through clinical trials NCT02921022, NCT02910882, and NCT02715804, only collagenase was only explored within this study. If the results of the present study bear out in humans, it may be that the degradation of collagen has a more important role to take in the ability to help deliver systematic agents into the tumor as compared to HA degradation.

In future studies, administration of both collagenase and hyaluronidase could be incorporated to determine the causal role of stromal content on interstitial hypertension and molecular uptake. This change in tumor stroma makeup in collagen/HA ratio could be fit with the linear elastic model used here, as has been shown before, and might lead to predictive power about how changes affect vascular patency. In this pursuit, ideally the intratumoral heterogeneity of collagen and hyaluronic acid, vascular patency, and molecular uptake should be investigated to determine spatial and temporal dynamics to optimize both molecular delivery and therapeutic treatment, and to assess how they change with collagen or HA degrading therapies. It is very likely that the temporal dynamics and the concentration dependencies could make the response of tumors to matrix degradation quite complex. Regrowth of blood vessels to allow increased perfusion to the tumor interior could likely take days, whereas the degradation occurs on the timescale of hours. So, working on ways to chronically administer enzymes and determine optimal degradation/revascularization times would be a useful study. Unfortunately the degradation of collagen studied here essentially liquified the collagen present in the tumor locally, making quantitative histology analysis of the changes nearly impossible. The methodology to track changes such as this must be done either with more subtle therapy or via some other assay.

Another line of important investigation would be to carry out this work in primary patient-derived cancer cell lines, often called PDX lines, and/or in humanized nude or NGS mouse models, to confirm the observations are similar to the human derived xenograft models used here. It is still unclear if the variation in morphology of PDT or humanized PDAC models would be different, but his investigation would be an important secondary step in this interpretation of the observed results.

## Conclusions

In summary, this study demonstrated that within PDAC tumors, TPP and SS^14^ are dominated by the collagen density, which varies considerably between locations both between and within each tumor. The analysis of pathology results from the same sites the pressure measurement helped illustrate these concepts. A biophysical spring model was applied to the data to fit the relationship of HA to collagen data, as has been shown previously. This shows that interstitial pressure and the saturating solid stress value, fit biological plausibility (p < 0.01), and that this data fits with expected norms for a first order linear elastic model. One important original discovery here, is that localized tumor measurements of patent vessel area fraction showed strong inverse power dependence between verteporfin uptake and TTP in both orthotopic and subcutaneous locations, without much difference between them. Active enzymatic degradation of interstitial collagen within the AsPC-1 tumors illustrated a reduction in all TTP values and a statistically significant increase in verteporfin delivery to the tumor. This study illustrates how the highly heterogeneous values of collagen build up, and the resulting complexity of the collagen makeup, influence local pressure and lead to decreases of perfused blood vessels. Appreciation and study of the microregional pressures induced by collagen and how they might be altered should lead to future discoveries in PDAC management.

## Electronic supplementary material

Supplementary Information
