# Supplementary material for: Collagen Complexity Spatially Defines Microregions of Total Tissue Pressure in Pancreatic Cancer
Source: Sci Rep. 2017 Aug 30;7:10093. doi: 10.1038/s41598-017-10671-w (PMC5577321; doi:10.1038/s41598-017-10671-w)
Supplement: Supplementary file 1 — Supplementary Information [file 41598_2017_10671_MOESM1_ESM.pdf]

Supplementary Information

For paper - Collagen Complexity Spatially Defines Microregions of Total Tissue Pressure in Pancreatic Cancer

Michael D. Nieskoski[1], Kayla Marra[1], Jason R. Gunn[1], P. Jack Hoopes[1,2], Marvin M. Doyley[3], Tayyaba Hasan[4], B. Stuart Trembly[1], Brian W. Pogue[1*]

[1]Thayer School of Engineering at Dartmouth, Hanover NH 03755

[2]Department of Surgery, Geisel School of Medicine at Dartmouth, Hanover NH 03755

[3]Department of Electrical and Computer Engineering, University of Rochester, Rochester NY

[4]Wellman Center for Photomedicine, Massachusetts General Hospital, Harvard Medical School, Boston MA 02114

*Corresponding Author: Brian.W.Pogue@Dartmouth.edu

Materials and Methods

Acquisition of total tissue pressure (TTP) and interstitial fluid pressure (IFP) in multiple locations within pancreatic cancer

Pressure measurements were performed using a Millar Mikrotip piezo-electric pressure catheter (model SPR-671, 0.47 mm diameter) with a dynamic pressure range from -50 mmHg to 300 mmHg (Millar.com) and a nominal sensitivity of 5μV/V/mmHg. The Millar pressure catheter has four ports, a positive and negative terminal for excitation voltage and a positive and negative terminal for output voltage. The output signal corresponds to the difference between the positive and negative terminal output voltages. The excitation voltage for the probe was supplied by an external voltage supply set at 5 V_{DC}. The output signal terminals of the pressure catheter were connected to an AD8221 precision instrumentation amplifier (Analog Device LLC). The programmable gain of the instrumentation amplifier was set to 991 using a 49.9 Ω 1% resistor placed across the gain terminal. The output of the instrumentation amplifier was passed through a first-order RC low pass filter with a cutoff frequency, f_c = 0.1 Hz. A low-pass filter was required to filter the 60 Hz electrical hum, which interfered with the signal output of the pressure transducer. The low-pass filter was connected to a Vernier LabPro data acquisition unit (Vernier Software and Technology) using Vernier voltage probes, +/- 10 V range. Data was recorded and analyzed using Venier LoggerPro3 software. The data acquisition rate was set to 60 samples per minute. An illustration of the setup and system diagram are shown in Figure S1.

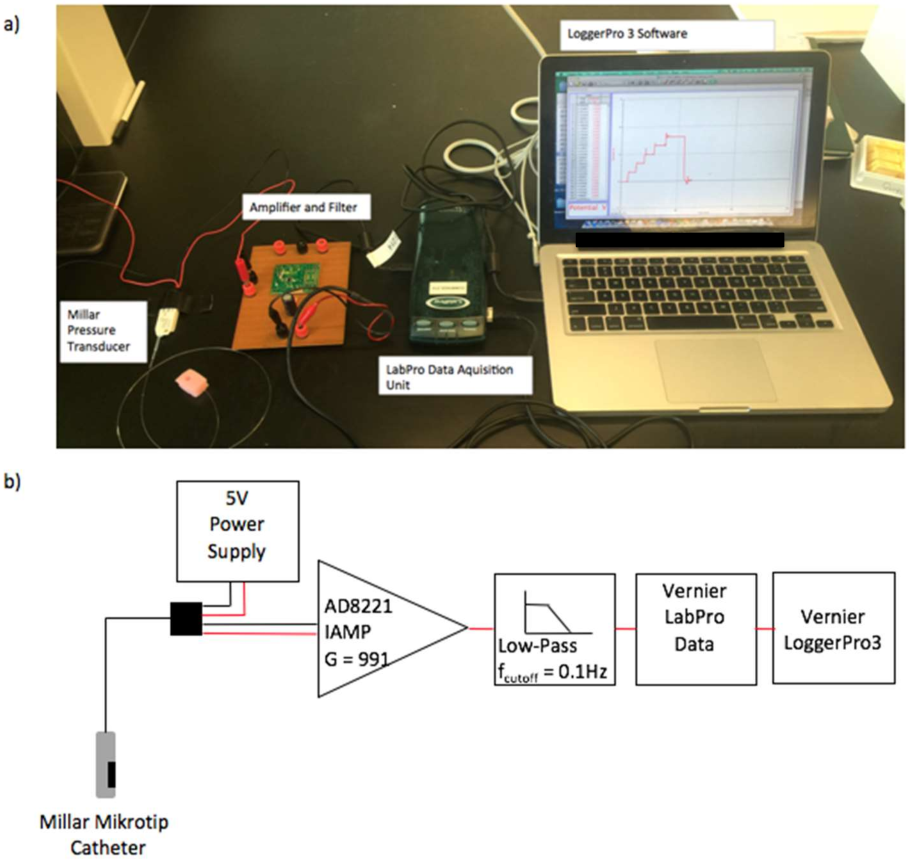

Figure S1. Image of pressure sensor system. The system includes a Millar Mikrotip pressure catheter (a) and the system diagram (b) includes an instrumentation amplifier, a first-order low pass filter, a Vernier LabPro data acquisition unit, and the Vernier LoggerPro3 software.

Three tumor locations were measured sequentially for each tumor; a single location for IFP and all three locations were measured for TTP. The tumor locations were separated by approximately 4 mm and were located along the same horizontal plane, illustrated in Figure S2. To place the pressure catheter into tumor tissue, a 23-gauge needle was first introduced into the tissue and the Millar catheter was quickly placed into the track created by the needle. The probe was removed and reinserted several times to avoid adherence to the surrounding tissue. The Millar sensor was approximately located 3 to 5 mm from the tumor surface. Data were collected for a minimum of 10 minutes for each tumor location or until a constant pressure value was observed.

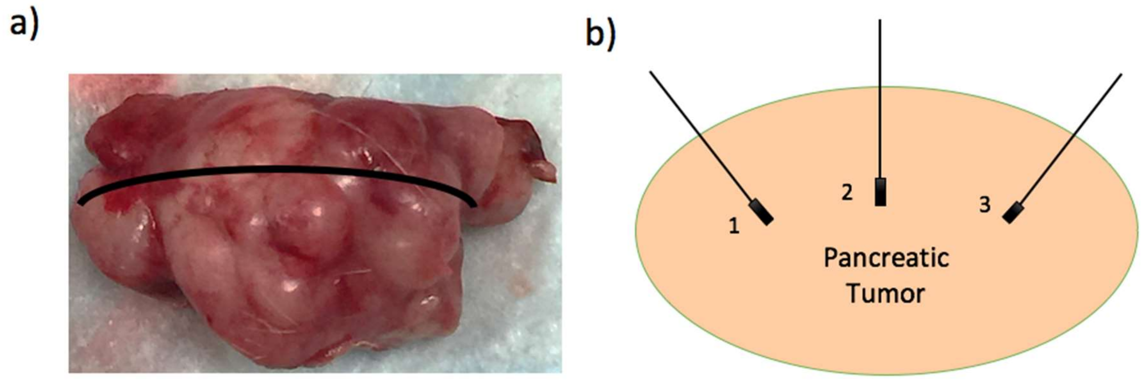

Figure S2. Total Tissue Pressure (TTP) measurements conducted within three locations within tumor interstitium**.** A horizontal plane was defined within each tumor analyzed (a). A Millar Mikrotip catheter (SPR-671) will be inserted into three locations, approximately 4 mm separated, along the same horizontal plane (b). The probe will be approximately 3 to 5 mm from the tumor surface.

To co-localize pressure measurements with histological analysis of collagen and hyaluronic acid, as well as vascular patency and verteporfin uptake, each tumor was excised and 23 gauge hypodermic needles was placed within the track of each pressure measurement. The tumor was placed into formalin for 24 hours. The hypodermic needles were retracted, placed into India ink, and re-inserted back into the needle track. This process is shown in Figure S3. The excised tumor was then cut along the horizontal plane containing the acquired pressure measurements and sent to clinical pathology to be stained using hematoxylin & eosin (H&E), Masson Trichrome stain (collagen), and hyaluronic acid binding protein-1 (hyaluronic acid).

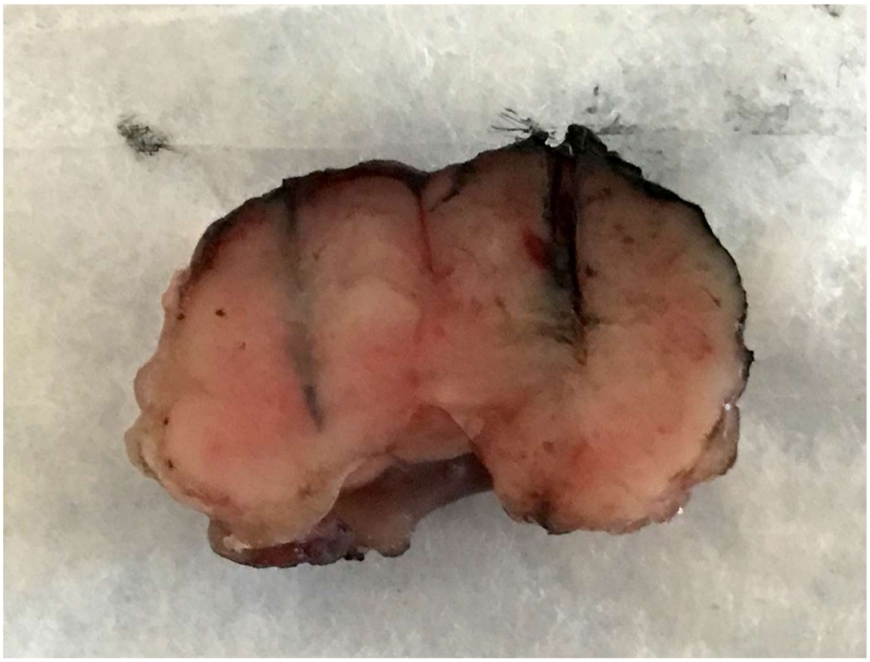

Figure S3. Localization of total tissue pressure (TTP) measurements in pancreatic tumors. Post-acquisition of TTP, 23 gauge hypodermic needles were placed within the location of each pressure measurement and the excised tumor was placed in formalin for 24 hours. The tumor was removed from formalin, the needles retracted from the tumor, placed in India Ink, and reinserted back into the needle tracks.

Color Segmentation of Masson Trichrome Stain

Tumor sections stained with Masson Trichrome were imaged at 10x using the Vectra 3 slide scanner and saved in 8-bit RGB format. The image was analyzed with color segmentation to extract collagen by converting RGB format to Hue-Saturation-Value (HSV) color space. Hue-Saturation-Value (HSV) is a three-dimensional representation of color which separates color intensity, z-plane, and chromaticity, x-axis and y-axis. Hue provides chromatic differences of dominant color planes, where red, yellow, green, cyan, blue, and magenta are represented by numerical values 0, 1/6, 1/3. 1/2, 2/3 5/6, respectively. The representation of Saturation is a radial position of colors ranging between white (Saturation = 0) to the dominant color representation (Saturation = 1). Value describes intensity or luminance and ranges between black (Value = 0) and white (Value = 1). Due to the two chromaticity axes, a change in hue is a more dominant shift in color identification than Saturation, therefore, hue will be used as a prominent variable to apply color segmentation to the Masson trichrome images.

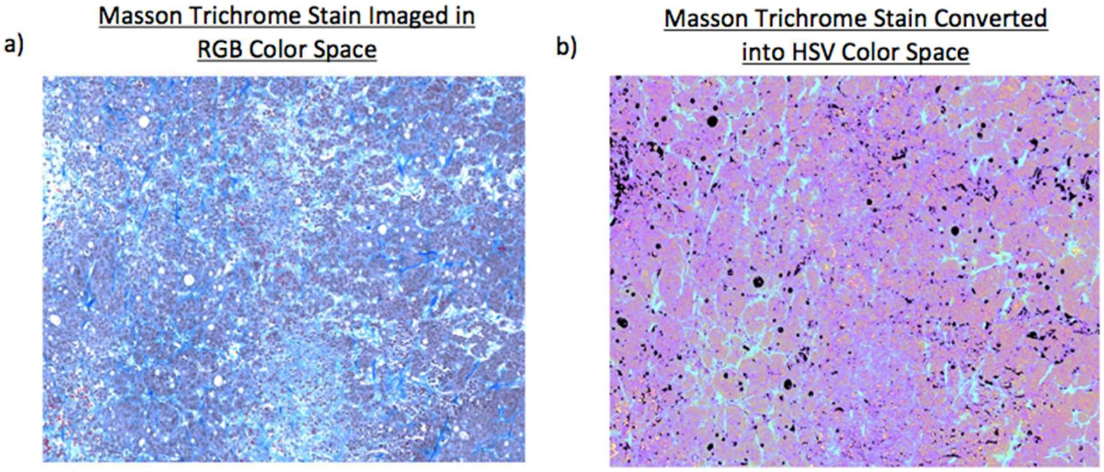

Figure S4. Masson Trichrome Stain imaged in RGB and HSV color space. Masson trichrome stain was imaged at 10x resolution using the Vectra 3 scanner. The image was viewed in RGB color space (a) and converted into HSV color space using MATLAB (b). The HSV color space clearly depicts collagen fibers throughout the tumor, while the RGB image illustrates difficulties in identifying individual collagen fibers.

Figure S4. illustrates the significance of the HSV color space for segmenting collagen fibers from the image. In Figure S4a, an 10x image of Masson Trichrome stain is shown in RGB format and converted to HSV color space using MATLAB rgb2hsv() function, Figure S4b. It is clear from these image that HSV more accurately depicts individual collage fibers within the tissue stain, by illustrating the collagen fibers and tissue background with distinct chromaticity difference. HSV color space represents blue by a value of 0.66 within the HSV color space. The filters chosen for color segmentation

were hue ranging between 0.6 and 0.7, saturation ranging between 0.7 and 1, and value equal to 1. By selecting these filters, slight difference in chromaticity plane for blue pixels will remain after the applied filter, producing two images representing collagen and the background, respectively. An illustration of this applied filter is shown in Figure S5.

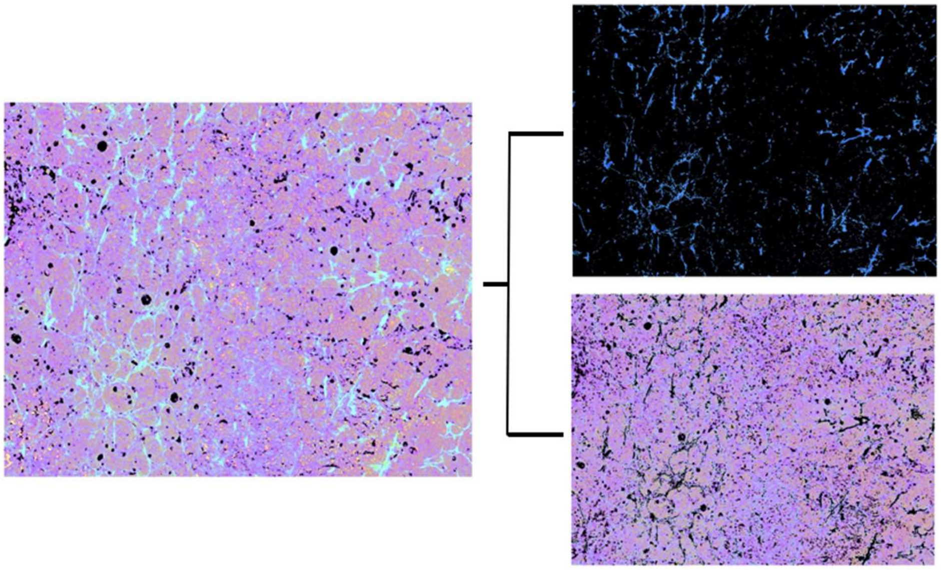

Figure S5. Color segmentation of Collagen within Masson Trichrome stain using HSV color space. A Masson Trichrome image was converted from RGB to HSV in MATLAB. An appropriate filter of Hue = 0.6 to 0.7, Saturation = 0.7 to 1, and Value = 1, was applied to the image to segment collagen pixels within the image.

Biomechanical Modeling of Collagen and Hyaluronic Acid in PDAC Tissue

The role of the collagen matrix within pancreatic tissue is to elastically resists the swelling force produced by HA, producing an equal stress to the Donnan equilibrium pressure to maintain a constant tissue volume. The solid collagen matrix counterbalances the large absorption pressure resulting from this osmotic swelling, thereby, limiting fluid absorption and achieving tissue homeostasis. Therefore, the pressure elevation is an indirect measurement of the internal stress imposed by the collagen-proteoglycan matrix.

The measurable mechanical properties of the tissue, including viscoelasticity and tumor stiffness, are strongly affected by enhanced deposition of collagen and HA within the ECM. A common biomechanical model used to describe viscoelastic tissue interactions is the standard solid model, which consists of a series combination of a spring (representing collagen elasticity) and a parallel combination of a spring and damper (representing HA). In this model, stress (σ) is applied to the system and the resulting strain (ε) is restricted by the elastic modulus of each spring and the damper, which is affected by the rate of deformation. In tumor tissue, steady state is assumed, therefore, the damping coefficient is removed and the system is solely dependent on the

series combination of springs, representing the elastic modulus of collagen and HA, respectively.

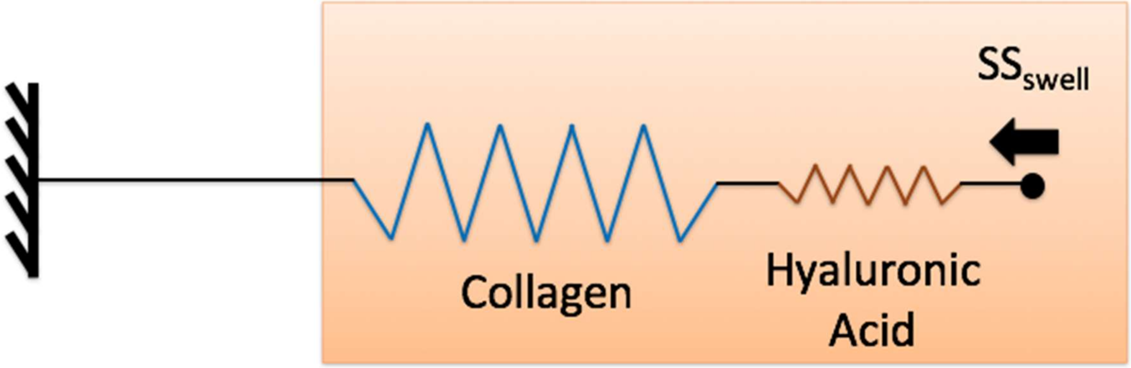

Figure S6. Modified standard solid model to illustrate solid stress (SS) within pancreatic tumors. This model consists of a series combination of springs, representing collagen and hyaluronic acid, respectively, and a stress term representing HA swelling stress.

Elevated osmotic pressure due to Donnan equilibrium pressure and electromechanical repulsion within HA results in internal swelling of the tissue matrix. Collagen elastically restricts this swelling force by providing a counter-balance to achieve homeostasis within pancreatic tissue. Therefore, this swelling stress can be represented as a net internal stress, SS_{swell}, within the standard solid model and the resulting interaction between collagen and HA in respect to SS is shown in Figure S6. By applying Hooke's law to this system, the stress applied to collagen within tissue can be represented as:

$$SS = SS_{swell}\frac{[C]}{[C]+[HA]}$$

$$SS = SS_{swell}\frac{1}{1+\frac{[HA]}{[C]}} \quad [1]$$

where [C] and [HA] represent collagen and HA content within tissue, respectively, and SS_{swell} represents the swelling stress imposed by HA.

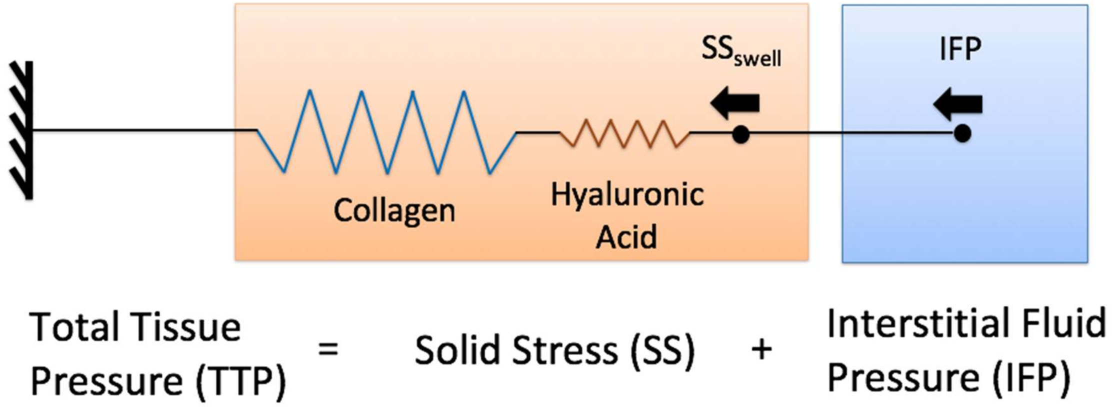

Figure S7. Modified standard solid model to incorporate solid stress and with the role of interstitial fluid pressure.

The inherent nature of a piezoelectric pressure catheter used in this study is that it measures all applied stress on the active element, thereby integrating together all TTP, which are thought to be a combination of both growth induced SS and IFP. Therefore, IFP must be added into this model to fully describe the total interactions of pressure within the tumor interstitial space. This work has shown that IFP is a measurable quantity, by either a modified piezoelectric sensor or the wick-in-needle technique. Therefore, the model in Figure S6 can be slightly modified to introduce a constant IFP source, as illustrated in Figure S7. The resulting elastic model can be solved resulting in the following equation.

$$TTP = SS_{swell}\frac{1}{1+\frac{[HA]}{[C]}} + IFP \qquad [2]$$

This model represents both SS arising from HA swelling stress applied to a series combination of springs, representing collagen and HA, respectively, and a constant IFP representing free fluid pressure within the tissue.

Texture Analysis on Collagen Distribution in Tumor Sections

Texture analysis was used to evaluate collagen heterogeneity in localized ROI's through quantification of entropy, uniformity, and fractal dimension. The goal was to determine whether irregularity and complexity of collagen distribution in localized regions can serve as an indicator for elevated tissue pressure and reduced molecular uptake within the tumor.

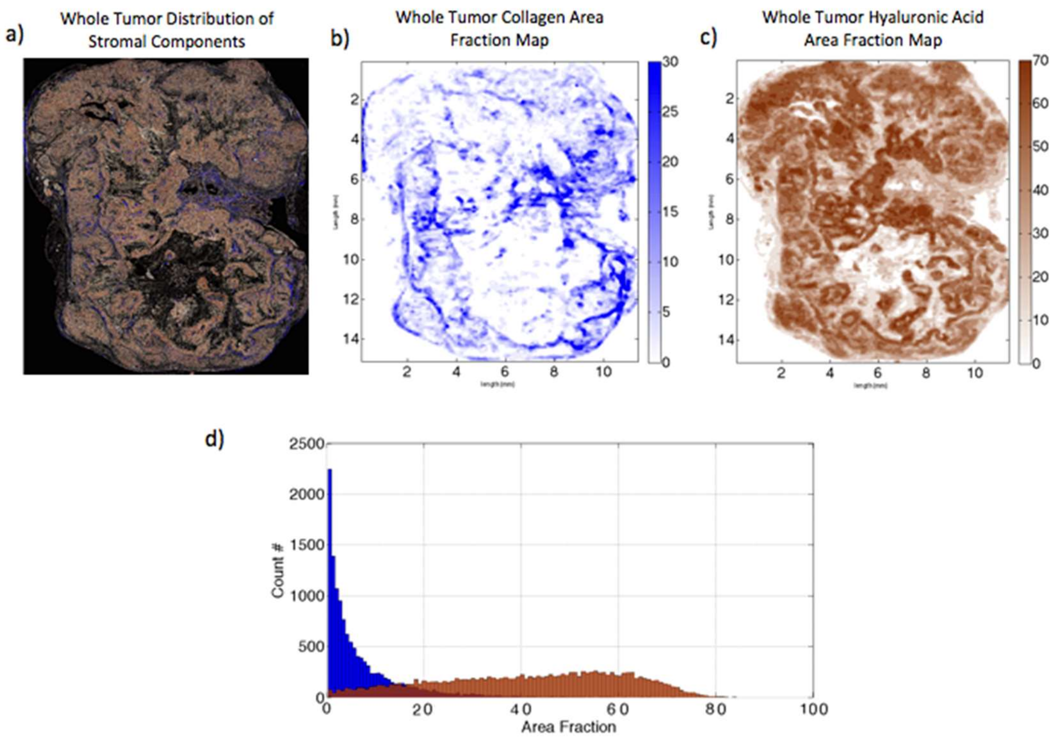

Figure S8. Whole tumor section area fraction map of collagen and hyaluronic acid. Masson Trichrome stain and HABP-1 stain were color segmented to represent collagen and HA, respectively, and co-registered to ensure spatial correlation (a). Area fraction maps were made for collagen (b) and HA (c). Histograms of collagen and hyaluronic acid area fractions are overlaid and displayed (d).

Histogram analysis was conducted to quantify both entropy and uniformity of collagen distribution within select ROI's within a tumor section. Entropy is a measure of irregularity in the distribution of collagen within tissue, while uniformity is a measure of how close the image is to a uniform distribution. To conduct this analysis, Masson Trichrome stain and HABP-1 stained images were color segmented to represent collagen and HA, respectively, and co-registered to ensure spatial correlation. The image was divided into 100 μm sections, in which the area fraction of collagen and HA was computed. The results were then displayed as two separate images representing whole tumor area fraction map of both collagen and HA, as shown in Figure S8. To compute entropy and uniformity, the following equations were applied to the histogram data:

$$\text{Entropy} = -\sum_{I=1}^{N}\big(P(V)\big)\log_2\big(P(V)\big) \qquad [3]$$

$$\text{Uniformity} = \sum_{I=1}^{N}[P(V)]^2 \qquad [4]$$

Where P(V) represents the probability of occurrence. As entropy increases and uniformity decrease, the collagen distribution within the tumor is more heterogeneous.

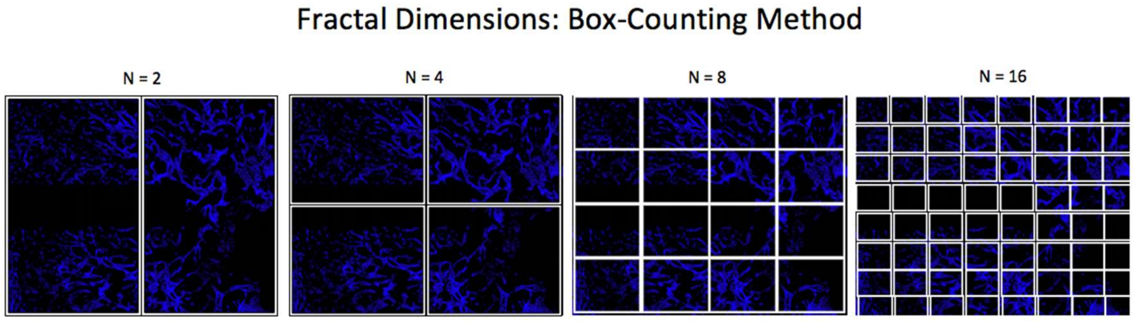

Figure S9. Fractal Dimensions Box Counting Method.

Fractal dimension is a quantitative method to define complexity of collagen distribution within tumors and the method applied in this work was the box counting method. The box-counting method is the most common method to compute fractal dimensions due to the simplicity of its methodology and ease to implement. This method consists of dividing an image segmented for collagen distribution into 2^i number of equal sized boxes, where i represents the iteration number, illustrated in Figure S9. The number of boxes containing collagen and the box size are recorded for each iteration. This process is continued until the number of boxes approximately equals the number of pixels within the image.

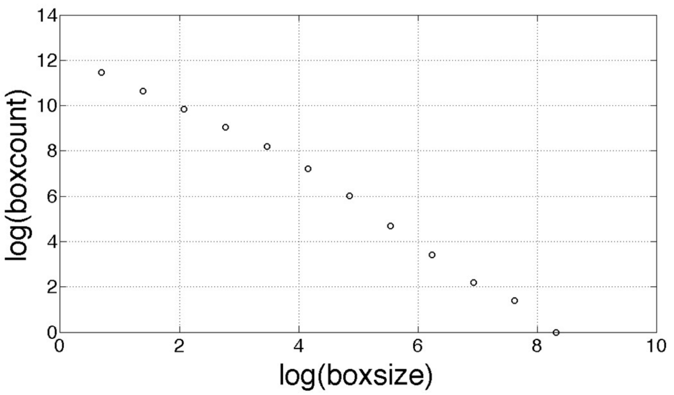

Figure S10. Computing Fractal Dimensions using the box counting method. The results of Figure 6.20 are then plotted by taking the log of the number of boxes, represented by boxcount, compared to the log of the box sizes. The slope of the resulting plot represents the fractal dimension of the collagen distribution within this tumor section.

The resulting data is plotted using the log of the number of boxes, compared with the log of the box size, as illustrated in Figure S10. Fractal dimension is computed as the slope of Figure S10, where the mathematical expression is:

$$\text{Fractal Dimension} = -\frac{\log(\text{boxcount})}{\log(\text{boxsize})} \qquad [5]$$

Fractal dimension provide information on the complexity of the collagen distribution

within pancreatic tumors. If the collagen within the tumor interstitial space consists of localized fibers, the fractal dimension would be smaller than collagen distribution that possesses a branch-like structure. The goal is to determine that increased complexity of collagen within tumors correlates to elevated TTP, as illustrated in Figure 5.

Estimated TTP heterogeneity within Pancreatic Tumor Tissue

The strong correlation between collagen and TTP reported in Figure 3a suggests localized collagen distribution could be used to approximate localized TTP. To illustrate this concept, collagen area fraction (%) maps of an entire tumor section were converted to TTP maps to illustrate localized heterogeneity within an entire tumor section. For each map, the tumor section was divided into several ROI's and the collagen area fraction was computed. The ROI were selected to squares with side lengths of 20μm, 50μm, 100μm, 200μm, and 500μm, as illustrated in Figure S8.

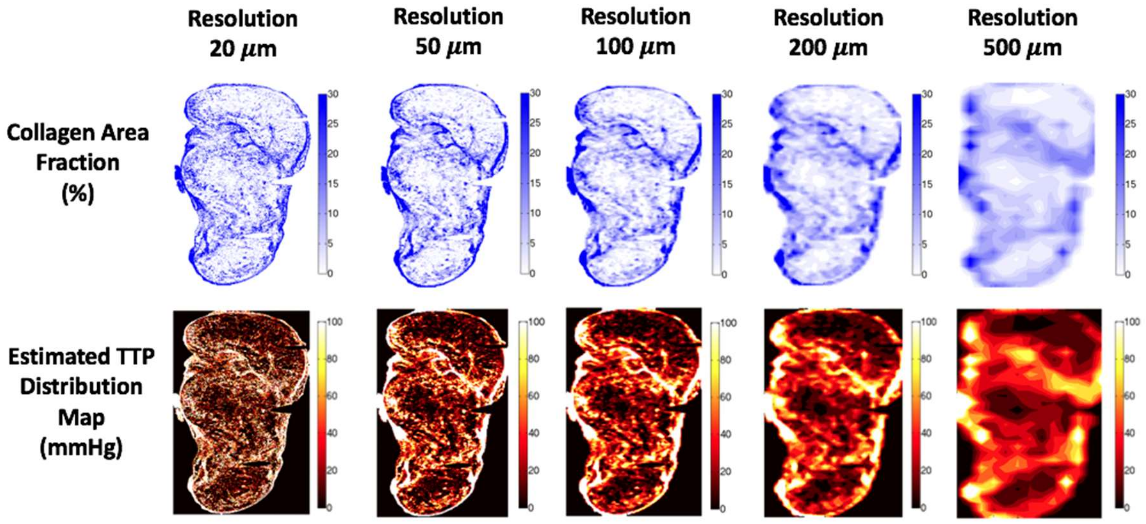

Figure S11. Estimated TTP with various resolution. The ROI selected to segment each tumor section were 20μm, 50μm, 100μm, 200μm, and 500μm. In each ROI, an area fraction of collagen was computed and related to TTP using the strong correlation reported in Figure 3a.

Figure S11 illustrates estimated TTP maps for an entire tumor section with various resolutions. As shown, an ROI of 20μm^2 correlates TTP to individual collagen while a larger ROI of 500μm^2 ignores the fibrous nature of collagen and outputs a more approximate regional TTP. Future analysis might investigate spatial relationships of collagen and TTP further to determine the accuracy of these approximated maps and illuminate the heterogeneity within pancreatic tumors.
